# Exploration of the Linkages between Lignin and Carbohydrates in Kraft Pulp from Wheat Straw Using a ^13^C/^2^H Isotopic Tracer

**DOI:** 10.3390/molecules28227493

**Published:** 2023-11-09

**Authors:** Hujun Niu, Xudong Chen, Yunbo Zhao, Junyi Zhou, Yimin Xie

**Affiliations:** 1 Research Institute of Pulp & Paper Engineering, Hubei University of Technology, Wuhan 430068, China; 102100479@hbut.edu.cn (H.N.); 102000415@hbut.edu.cn (X.C.); 102100481@hbut.edu.cn (Y.Z.); 102110526@hbut.edu.cn (J.Z.); 2 Hubei Provincial Key Laboratory of Green Materials for Light Industry, Hubei University of Technology, Wuhan 430068, China

**Keywords:** wheat straw, lignin–carbohydrate complex, isotope tracer, kraft pulping, ionic liquid

## Abstract

To further our understanding of the change in association between lignin and carbohydrates after kraft pulping, isotope-labeled kraft pulp (KP) was prepared using ^13^C and D double-isotope-labeled wheat straw, and it was subjected to enzymatic hydrolysis and ionic liquid treatment to explore the linkages between lignin and carbohydrate complexes in wheat straw. Isotope abundance determination showed that ^13^C and D abundances in the experimental groups were substantially higher than those in the control group, indicating that the injected exogenous coniferin-[α-^13^C], coniferin-[γ-^13^C], and d-glucose-[6-D_2_] were effectively absorbed and metabolized during wheat internode growth. Solid-state CP/MAS ^13^C-NMR spectroscopy showed that lignin was mainly linked to polysaccharides via acetal, benzyl ether, and benzyl ester bonds. Kraft pulp (KP) from the labeled wheat straw was degraded by cellulase. The obtained residue was fractionated using the ionic liquid DMSO/TBAH to separate the cellulose–lignin complex (KP-CLC) and xylan–lignin complex (KP-XLC). X-ray diffractometer determination showed that the KP-CLC regenerated cellulose type II from type I after the ionic liquid conversion. The ^13^C-NMR spectrum of Ac-En-KP-CLC showed that the cellulose–lignin complex structure was chemically bonded between the lignin and cellulose through acetal and benzyl ether bonds. The ^13^C-NMR spectrum of En-KP-XLC showed a lignin–hemicellulose complex structure, wherein lignin and xylan were chemically bonded by benzyl ether and acetal bonds. These results indicate that the cross-linking between lignin and carbohydrates exists in lignocellulosic fibers even after kraft pulping.

## 1. Introduction

Based on the global supply of wood and China’s current situation, some researchers believe that wheat straw should be effectively used in pulping and papermaking [1,2]. The chemical components of different wheat varieties are different; yet, on average, wheat straw consists of 33–40% cellulose, 20–25% hemicellulose, 15–20% lignin, 2–7% ash, 5% extractives, and a little pectin. [3] The strong chemical bonds between lignin, cellulose, and hemicellulose in plant fibers result in a large amount of energy and chemical consumption in the pulping process, as well as in the subsequent bleaching process [4,5]. Therefore, it is of great practical importance to understand the bonding mode between lignin and carbohydrates for efficiently separating lignin, cellulose, and hemicellulose [6,7].

In order to further study the connection between lignin and carbohydrates, Lundquist et al. [8] isolated 0.3% carbohydrates from the purified spruce milled wood lignin (MWL). The MWL was further treated with NaOH to reduce the carbohydrate content. It was found that spruce MWL contained various carbohydrates in the original hemicellulose. Half of the carbohydrates were dissolved after alkali treatment, and the dissolution of xylose was most significant. Johnson et al. [9] investigated the LCC isolated from Canadian poplar. At first, the wood mill was treated with an explosion method. Then, the product was extracted using ethyl acetate and precipitated using ether, and finally, four kinds of LCC were isolated via gel filtration. Scalbert et al. [10] isolated two LCC components from milled straw powder lignin (LM) and enzymatic lignin (LE), mainly containing xylose, followed by arabinose and glucose, though the lignins also contained a certain amount of uronic acid. Iversen et al. [11] investigated the isolation of various sugar components in the residual lignin of pine kraft pulp. They converted the sugar into sugar alcohol acetate and analyzed the contents and degrees of polymerization of various sugar alcohols via gas chromatography. The results showed there was a higher content of glucose in the residual lignin than other sugars, indicating that the residual lignin not only connected with hemicellulose but also bonded to cellulose. The carbohydrates in the LCC structure were linked to the carbon atoms of residual lignin in the side chain of the α-, β-, γ-position by ether bonds. These linkages are very stable in alkaline cooking. There, it is necessary to analyze in detail the LCC linkages in residual lignin in kraft pulp, especially, the bond between lignin moieties and cellulose.

Kraft pulping is the most important chemical pulping method, and it can efficiently remove most lignin from wood [12,13]. However, after kraft cooking, residual lignin is still present in the pulp [14,15]. This is mainly due to the condensation structure formed between the lignin moieties and the residue of the lignin–carbohydrate complexes in the fiber material, which severely hinders the selective separation of wood components [16]. The separation of residual lignin from the pulp can reveal the principle of delignification in kraft pulping and the chemical linkage information of lignin–carbohydrate complexes (LCCs) remaining in the pulp. Balakshin et al. [12] analyzed and compared the structures of residual and dissolved lignin isolated from pine kraft pulping using heteronuclear single quantum coherence (HSQC) nuclear magnetic resonance (NMR) technology and clarified the reaction mechanism in kraft pulping and the reactivity of lignin. Lu et al. [17] used ^13^C and ^2^H to label the lignin and carbohydrates of gymnosperms to investigate the chemical structure of residual lignin in kraft pulp (KP). The results showed that the acetal and benzyl ether bonds in the LCC of ginkgo fibers were stable during kraft cooking. These studies are crucial for understanding the mechanism of effectively removing lignin during pulping [18,19].

In the study of lignin structure, ^13^C, ^2^H, ^18^O, and ^31^P isotopes are increasingly favored by researchers because of their safety and absence of radiation [20]. Xie et al. [21] performed selective ^13^C enrichment of lignin side chains Cα, Cβ, and Cγ of ginkgo wood (*Ginkgo biloba* L.), and they synthesized lignin precursors coniferin-[α-^13^C], coniferin-[β-^13^C], and coniferin-[γ-^13^C]. The ^13^C abundance of the newly formed xylem was measured, and the Cα-enrichment was 3.5 times than that of natural abundance. Milled wood lignin (MWL) was prepared from the xylem of ginkgo wood, and its ^13^C-NMR spectrum was determined. The results showed that the ginkgo lignin structure included α-carbonyl, α-aldehyde, Cγ-carbonyl, Cγ-carboxyl, methylene, and phenyl coumarin. Hafrén et al. [22] added coniferin-[β-^13^C] and coniferin into the differentiated xylem of spruce and obtained cell-wall dehydrogenation (CW-DHP) after culture. The results showed that the treatment with coniferin-[β-^13^C] did not affect the biosynthesis of CW-DHP, and the information of β-O-4, β-β, β-5, and β-1 substructures in CW-DHP was quantitatively obtained using solid-state ^13^C-NMR. Xie et al. [23] also synthesized coniferin-(α-^13^C), coniferin-(β-^13^C), and coniferin-(γ-^13^C), and injected them into ginkgo plants to obtain ^13^C-rich LCC. Moreover, enzyme-degraded LCC (EDLCC) with a high lignin content was obtained via enzymatic degradation. Analysis using ^13^C-NMR showed that ether, ester, and ketal bonds were present in the side chain Cα position of lignin structural units in LCC, but no lignin–carbohydrate bond was found at Cβ and Cγ. 

In the present work, the aim is to study the association between lignin and carbohydrates, especially between lignin and cellulose after kraft pulping. The bond type and crosslinking position are investigated in detailed. Sodium acetate-[1-^13^C] and malonic acid-[1,3-^13^C] were used as starting materials for the synthesis of coniferin-[α-^13^C] and coniferin-[γ-^13^C], respectively. The ^13^C-labeled lignin precursors and D(deuterium)-labeled glucose were injected into the internode cavities in growing wheat stalks. After maturation, the wheat straw was subjected to kraft cooking for delignification to obtain KP rich in cellulose–lignin complexes (CLCs) and xylan–lignin complexes (XLCs). The pulp was fractionated using a DMSO/TBAH ionic liquid to obtain a cellulose–lignin complex (KP-CLC) and a xylan–lignin component (KP-XLC). The KP-CLC and KP-XLC were enzymatically hydrolyzed and acetylated. Finally, ^13^C-NMR and ^1^H-NMR were used to characterize the chemical connection in the CLC and XLC.

## 2. Results and Discussion

### 2.1. Treatment of Wheat Stalks with Lignin and Polysaccharide Precursors

**Figure 1:**
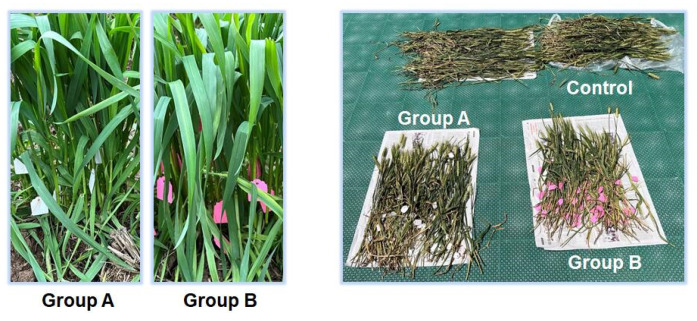
The administration of precursors of wheat 596 stalks and when fully matured. Note: Group A was a growing wheat injected with coniferin-[α-^13^C], d-glucose-[6-D_2_], and 4CL inhibitor; group B was a growing wheat injected with coniferin-[γ-^13^C], d-glucose-[6-D_2_], and 4CL inhibitor. The right side is the mature wheat after 20 days of natural growth.

**Figure 2:**
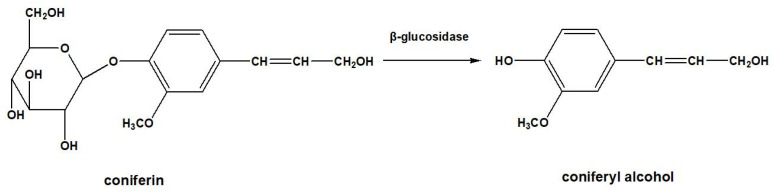
Formation of coniferyl alcohol.

Figure 1 shows the growth of wheat 596 (*Triticum aestivum* L., sp. 596). The wheat in group A and group B grew well after administration of precursors, and there was no significant difference compared with the control group [24,25,26]. In plant cells, β-glucosidase hydrolyzes coniferin-[α-^13^C] and coniferin-[γ-^13^C] to the corresponding coniferyl alcohol-[α-^13^C], coniferyl alcohol-[γ-^13^C] in the cell wall during lignification process, as shown in Figure 2. They are also involved in lignin biosynthesis in growing wheat internode cells [27]. The formation of coniferyl alcohol ensures the feasibility of subsequent research on the LCC and its characterization.

### 2.2. Analysis of ^13^C/^2^H Abundance in Wheat Internode Tissues

As shown in Table 1 and Table 2, δ^13^C and δD values in the experimental groups were substantially higher than those in the control group. In group A, the ^13^Cα/^12^Cα ratio of wheat straw labeled with ^13^Cα reached 2.604%, which was approximately 2.5 times that of the natural isotope ratio of wheat straw in the control group. In group B, the ^13^Cγ/^12^Cγ ratio of wheat straw labeled with ^13^Cγ reached 3.089%, which was approximately 2.8 times that of the control group. After D isotope labeling, the D6/H6 ratio of wheat straw in group A reached 0.280%, which was approximately 17.5 times than that of the control group. In group B, the D6/H6 ratio of wheat straw reached 0.370%, which was approximately 23 times that of the natural isotope ratio of the control. The results showed that exogenous coniferin-[α-^13^C], coniferin-[γ-^13^C], and d-glucose-[6-D_2_] were effectively absorbed and metabolized during the growth of wheat internode tissues; therefore, the lignin in the cell wall of wheat straw was labeled with ^13^C and the polysaccharide was labeled with D, which ensured the feasibility of subsequent research on the structure of the lignin–carbohydrate complex.

### 2.3. CP/MAS ^13^C-NMR Analysis of Wheat Straw Powder

**Figure 3:**
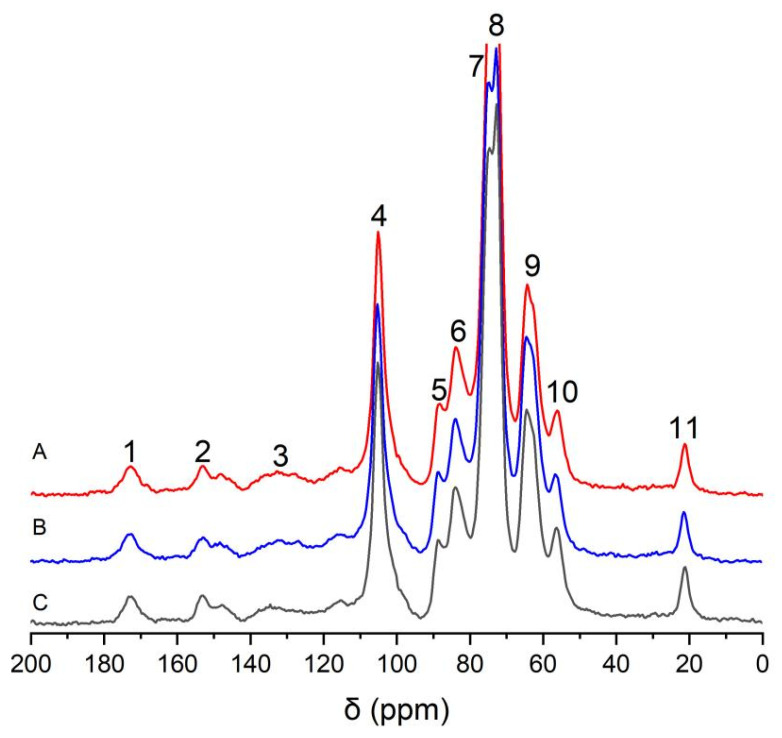
CP/MAS ^13^C-NMR spectra of wheat internodes (A: wheat straw injected with coniferin-[α-^13^C]; B: wheat straw injected with coniferin-[γ-^13^C]; C: control group).

Figure 3 shows the solid-state CP/MAS ^13^C-NMR spectra of the wheat straw samples. The solid-state ^13^C-NMR spectra of wheat straw administrated with coniferin-[α-^13^C] and coniferin-[γ-^13^C] and natural wheat straw were compared. The results showed that there was no significant change in the aromatic region (δ 110–160 ppm). This result strongly confirmed that coniferin-[α-^13^C] and coniferin-[γ-^13^C] could be normally converted into lignin during wheat internode tissue metabolism, ensuring wheat straw lignification, which was consistent with previous research results.

**Figure 4:**
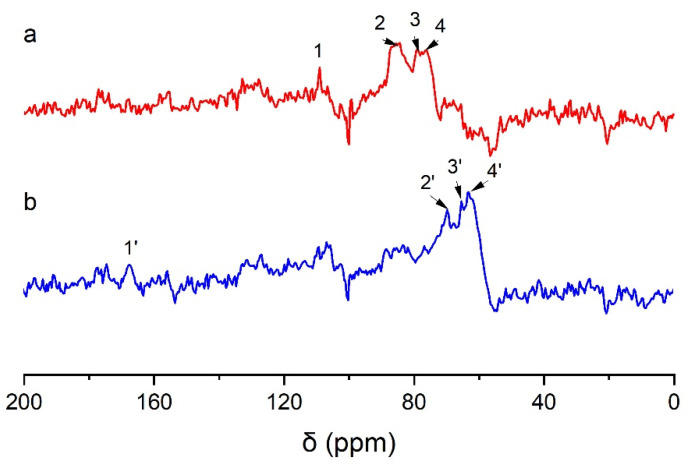
CP/MAS ^13^C-NMR differential spectra of wheat internode tissues (a: α-^13^C-labeled spectrum minus unlabeled spectrum, b: γ-^13^C-labeled spectrum minus unlabeled spectrum).

To distinguish between the CP/MAS NMR spectra of wheat straw labeled with α-^13^C and γ-^13^C and that of the control, differential spectra were prepared based on the same methoxy group content, as shown in Figure 4. The specific chemical shift assignments are presented in Table 3.

In Figure 4(a), the enhanced signal at a chemical shift of δ 109.0 ppm (No. 1) is assigned to Cα in the lignin linked to the polysaccharide by an acetal bond [28]. There is a strong and wide resonance peak at a chemical shift of 82–88 ppm (No. 2), and this signal is assigned to Cα in β-5, β-β, and moieties linked to carbohydrates by a benzyl ether bond [29]. The strong resonance signal at 76.4 ppm (No. 3) is assigned to Cα in the lignin linked to the polysaccharide by a benzyl ester bond. The signal at 74.6 ppm (No. 4) corresponds to the Cα of the β-O-4 structure [21]. In Figure 4(b), a weak signal peak at 167.3 ppm (No. 1’) is assigned to Cγ in ferulic acid derivatives in wheat straw. A sharp peak at 72.6 ppm (No. 2’) is assigned to the Cγ in the structure of pinoresinol (β-β). The signal at 64.4 ppm (No. 3’) is Cγ in the β-5 lignin structure. The signal at 62.5 ppm (No. 4’) arose from Cγ in the β-O-4 and β-1 structures of lignin.

**Figure 5:**
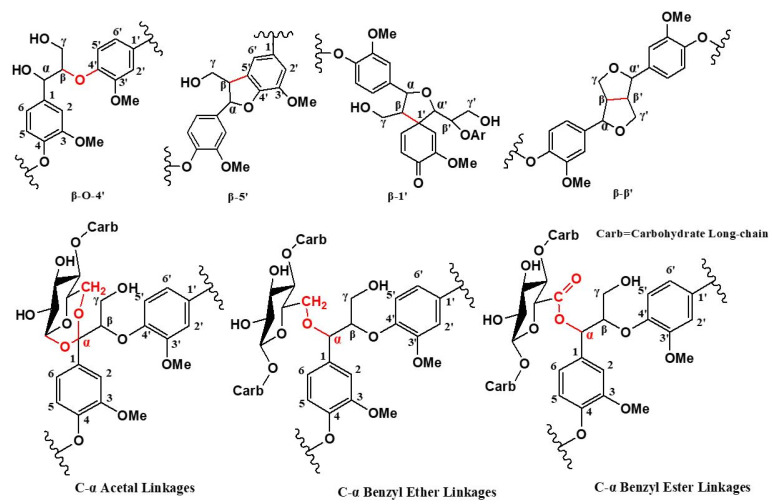
Linkages between lignin structural units and lignin–carbohydrate complexes.

The comparison of the results of the above differential spectra provided strong evidence of the presence of the following structures: β-aryl ether (β-O-4), phenylcoumarol (β-5), pinoresinol (β-β), β-1, and LCC in wheat straw, which laid a foundation for the subsequent study of the structure of CLC and XLC. The above results prove that phenylpropane moieties in the protolignin of wheat stalk are linked to carbohydrates at both α- and γ-positions. Figure 5 shows the possible connection types between lignin units and between lignin and carbohydrates.

### 2.4. XRD Characterization of KP-CLC and KP

**Figure 6:**
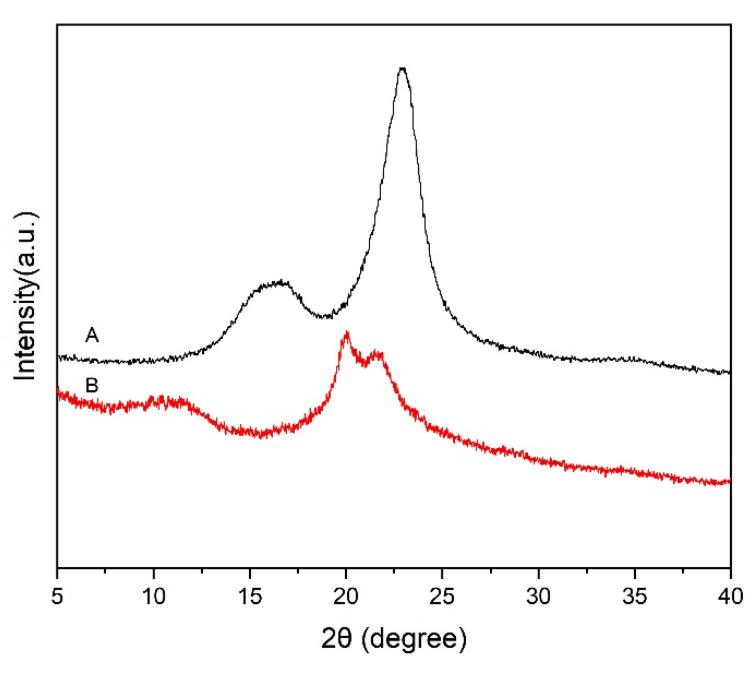
XRD spectra of the samples (A: wheat straw KP; B: KP-CLC).

As shown in Figure 6(A), wheat KP had clear XRD diffraction peaks at 2θ = 15.4°, 16.6°, and 22.8°, which can be assigned to 101, 10-1, and 002 crystal planes of cellulose type I, respectively. As shown in Figure 6(B), after dissolution and regeneration via ionic liquid DMSO/TBAH, KP-CLC exhibited sharp XRD diffraction peaks at 2θ = 20.0° and 21.9°, which were consistent with the characteristic diffraction angles of the crystal planes of cellulose type II, indicating that the residual crystalline cellulose in the pulp was transformed from cellulose type I to type II. The results also showed that the cellulose in the KP-CLC of wheat KP contained a crystalline region, and crystalline cellulose was present in the KP-CLC.

### 2.5. Chemical Structure Analysis of Ac-En-KP-CLC

#### 2.5.1. ^13^C-NMR Characterization of Ac-En-KP-CLC

**Figure 7:**
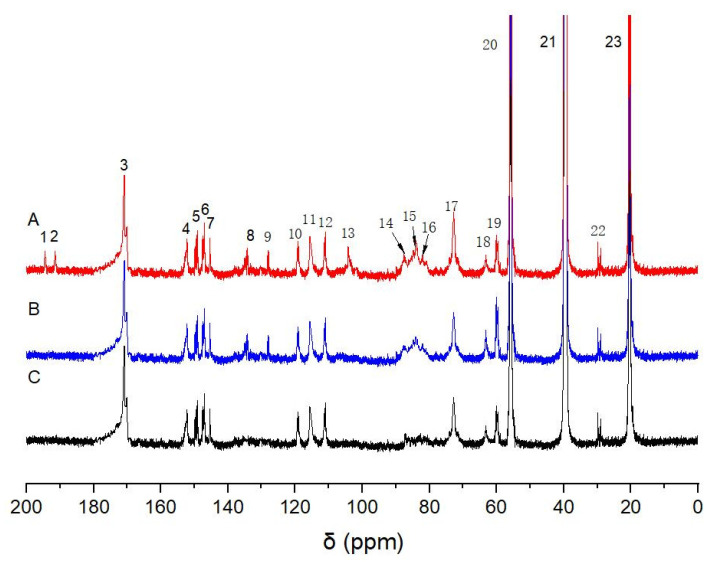
^13^C-NMR spectra of acetylated products of enzymatic degraded kraft pulp cellulose–lignin component (Ac-En-KP-CLC) (A: ^13^Cα/6-D labeled Ac-En-KP-CLC; B: ^13^Cγ/6-D labeled Ac-En-KP-CLC; C: unlabeled control samples).

As shown in Figure 7, the signals from the polysaccharides in the En-KP-CLC fraction were weakened after degradation by cellulase and hemicellulase. The stable methoxy peak at δ 55.5 ppm (No. 20) was used as the internal reference. The enhanced chemical signals at δ 194.5 ppm (No. 1) and 191.5 ppm (No. 2) are mainly assigned to α-CHO [30]. The signal at δ 170.8 ppm (No. 3) originated from the C=O bond in the acetyl group. Owing to the formation of an α-acetal bond between the lignin side chain Cα and cellulose, the peak at δ 104.2 ppm (No. 13) was substantially enhanced after ^13^Cα labeling. The enhanced signal with δ 87.6 ppm (No. 14) was assigned to the Cα of the lignin phenylcoumaran structure. The signal at δ 84.0 ppm (No. 15) can be assigned to the enhanced Cα of the pinoresinol structure. The signal at δ 81.9 ppm (No. 16) was enhanced via ^13^Cα labeling and assigned to the lignin side chain Cα linked to cellulose by a benzyl ether bond. The enhanced signal at δ 72.8 ppm (No. 17) can be assigned to the Cα in the lignin β-aryl ether structure. After ^13^Cγ labeling, in spectrum B, the peaks at δ 63.1 ppm (No. 18) and δ 60.8 ppm (No. 19) were enhanced compared to the control group. Therefore, the signals at No. 18 and No. 19 were assigned to Cγ in pinoresinol, β-1, and β-aryl ether structures. These results indicate that the residual lignin is bonded to cellulose by α-acetal and benzyl ether bonds after kraft pulping. However, no LCC signal is observed at γ-position of the phenylpropane moieties in the residual lignin. This reveals that the LCC linkages at γ-position are cleaved during kraft pulping.

#### 2.5.2. ^1^H-NMR Characterization of Ac-En-KP-CLC 

The Ac-En-KP-CLC of group A with Cα/6-D labeling, group B with Cγ/6-D labeling, and control group C were determined using ^1^H-NMR. The proton on the aromatic ring of lignin at δ 7.21 ppm was used as the internal reference to analyze the differential spectrum.

**Figure 8:**
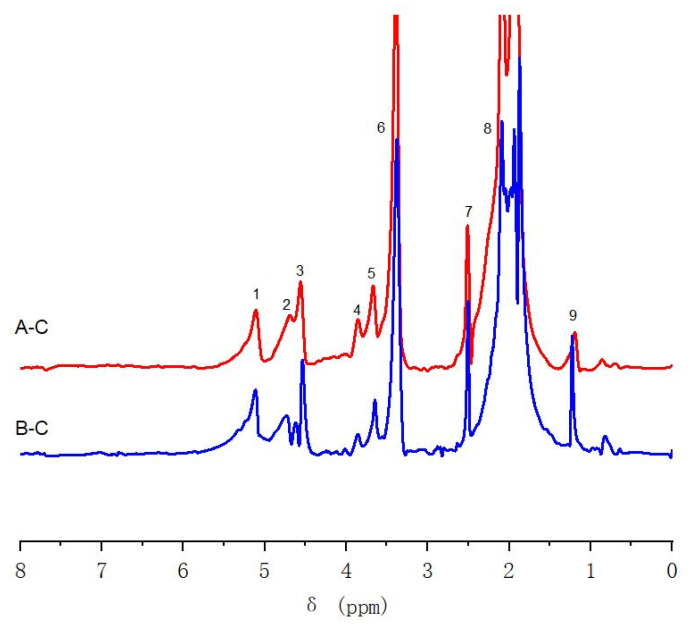
^1^H-NMR differential spectra of Ac-En-KP-CLC samples. Notes: A–C, Group A minus Group C; B–C, Group B minus Group C.

As shown in Figure 8, the signal at δ 5.12 ppm (No. 1) comes from acetylated α-H of lignin–lignin linkage, including β-O-4, β-5, and β-1 structures. The signals at δ 4.69 ppm (No. 2) and δ 4.53 ppm (No. 3) were derived from the 6-H and 6-H’ in the cellulose linked to lignin by the benzyl ether bond [31]. Combined with the ^13^C-NMR analysis of Ac-En-KP-CLC, the signal of the ester bond between lignin and cellulose was not observed in the ^1^H-NMR differential spectra, because these samples were acetylated beforehand. The signal at δ 3.85 ppm (No. 4) originated from the H on the methoxy group. The signal at δ 3.64 ppm (No. 5) originated from free H and H’ on C-6 of cellulose [32]. The signal at δ 3.37 ppm (No. 6) and 2.53 ppm (No. 7) originated from the water and solvent of the deuterated DMSO reagent, respectively. The signal at δ 1.8–2.1 ppm (No. 8) originated from the H on the acetyl group. The signal at δ 1.2 ppm (No. 9), originating from the highly shielded aliphatic H. From cellulose side, it is also proven that the C-6 of cellulose is connected with residual lignin by the benzyl ether bond even after kraft pulping.

### 2.6. Chemical Structure Analysis of En-KP-XLC 

#### ^13^C-NMR characterization of En-KP-XLC

**Figure 9:**
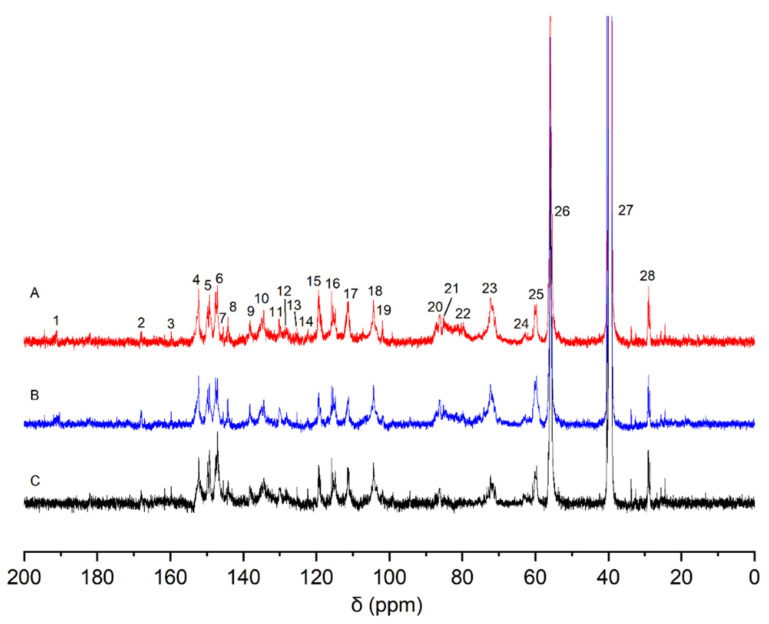
^13^C-NMR spectrum of En-KP-XLC (A: ^13^Cα/6-D labeled En-KP-XLC; B: ^13^Cγ/6-D labeled En-KP-XLC; C: unlabeled control samples).

Figure 9 shows the ^13^C-NMR spectra of the lignin–xylan complexes in KP after enzymatic hydrolysis (En-KP-XLC). To facilitate the assignment of signals from Cα and Cγ in the En-KP-XLC, a stable signal of methoxy at 56.1 ppm (No. 26) was used as the internal reference. The signal at δ 167.9 ppm (No. 2) originated from the C=O bond of the aromatic acid. The signal at δ 152.2 ppm (No. 6) originated from C3/C3’ in the lignin etherified 5-5’ structure. In the aromatic region δ 110–120 ppm (No. 15-No. 17), the differences between groups A, B, and C were not significant. They mainly originated from C2, C5, and C6 in the lignin benzene ring. At δ 101.8 ppm (No. 19), the signal intensity of group A was enhanced after ^13^Cα labeling and assigned to the Cα of the lignin side chain connected to xylan by an acetal bond. The δ 85 ppm (No. 20) and 86 ppm (No. 21) were assigned to the resonance of Cα in β-5 and β-β substructures in lignin. The signal at δ 81.4 ppm (No. 22) was assigned Cα of the side chain of the labeled lignin with an ether bond to the xylan [33]. The δ 72.3 ppm (No. 23) was derived from the Cα of lignin, the β-aryl ether structure [34]. The resonance signal at δ 62.9 ppm (No. 24) came from the Cα of the lignin β-1 substructure; furthermore, it could also be assigned to Cγ in β-5 and β-1 in lignin of group B. The signal at δ 60.2 ppm (No. 28) was from Cγ of the β-aryl ether structure. In summary, there was lignin side chain Cα in the En-KP-XLC that linked the LHC structure of xylan via acetal and ether bonds. These results indicate that there were an α-acetal linkage and α-ether bond still between lignin and xylan in the lignin–xylan complexes after kraft pulping. 

Xylan is biosynthesized in plants by converting d-glucose to glucuronic acid, which is subsequently converted into xylan through a series of reactions [35,36]. In this process, the D at the C-6 position of d-glucose-[6-D_2_] is hydrolyzed and eliminated; therefore, the ^1^H-NMR of the ^13^Cα/6-D labeled En-KP-XLC was not studied.

## 3. Experiment

### 3.1. Materials 

Wheat Emai 596 (*Triticum aestivum* L. sp.596) was provided by Hubei Academy of Agricultural Sciences (Wuhan, China). Sodium acetate-1-^13^C and d-glucose-[6-D_2_] were purchased from Sigma-Aldrich (St. Louis, MO, USA). The 3,4-methylenedioxycinnamic acid (a 4CL inhibitor) was purchased from Aladdin Reagent (Shanghai, China). All the other chemicals were of analytical grade.

### 3.2. Methods

#### 3.2.1. Synthesis of Isotope-Labeled Lignin Precursors

**Figure 10:**
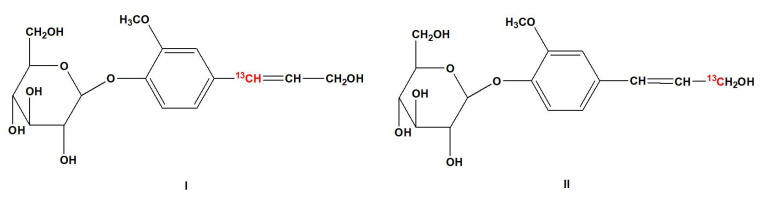
Chemical structures of coniferin-[α-^13^C] (**I**) and coniferin-[γ-^13^C] (**II**).

Based on the method described by Xie et al. [21], sodium acetate-1-^13^C and malonic acid-[1,3-^13^C] were used as starting materials for the synthesis of coniferin-[α-^13^C] and coniferin-[γ-^13^C]. The chemical structures of coniferin-[α-^13^C] and coniferin-[γ-^13^C] are shown in Figure 10. The yield of coniferin-[α-^13^C] was 7.5% and the melting point was 184.5 °C. The yield of coniferin-[γ-^13^C] was 35.0% and the melting point was 185 °C.

#### 3.2.2. Administration of Wheat Stalks with ^13^C and D Double-Isotope-Labeled Precursors

Emai 596 was selected as the biologically cultured wheat sample. In early April, the solution composed of lignin precursor coniferin-[α-^13^C] or coniferin-[γ-^13^C], together with polysaccharide precursor d-glucose-[6-D_2_] (3.33 mg/mL), and the 4CL inhibitor (4 mg/mL) (Table 4) were injected into the stalk cavities of internodes 1, 2, 3, and 4 with a 1 mL sterile micro syringe, starting from the first section of the wheat root in groups A and B. Every time, 0.5 mL reagent was injected, once every 5 days, and the injection was completed within 20 days. Because the isotope-labeled precursors are very expensive and the wheat growth cycle is long, we did not repeat the experiment. In order to reduce the error of the experiment, 100 wheat plants were processed in each experimental group. The control group did not receive any treatment. After injecting, the plants were allowed to grow for another 20 days for wheat heading and maturity.

#### 3.2.3. Preparation of Wheat Straw Powder

**Figure 11:**
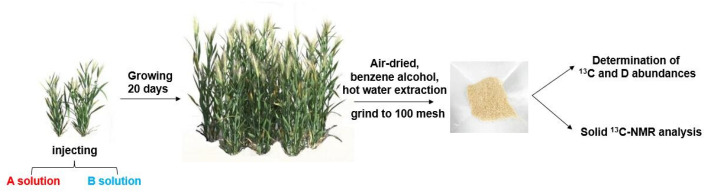
Injection of precursors to wheat stalk (A solution: coniferin-[α-^13^C], d-glucose-[6-D_2_], and 4CL inhibitors; B solution: coniferin-[γ-^13^C], d-glucose-[6-D_2_], and 4CL inhibitors).

The mature wheat stalks that fully absorbed the two lignin precursors labeled ^13^Cα and ^13^Cγ and polysaccharide precursor d-glucose-[6-D_2_] were harvested. The internode tissues of the wheat were milled into a 60–80 mesh powder using a Weily mill after being fully air-dried. The powder was then thoroughly extracted using an ethanol/benzene mixture (1/2, *v*/*v*) and hot water. The basic process of preparation and subsequent treatment of the straw powder is shown in Figure 11.

#### 3.2.4. Determination of ^13^C and D Abundances

A total of 5 mg of wheat straw samples from the control group, group A labeled with ^13^Cα and D, and group B labeled with ^13^Cγ and D was used for ^13^C isotope abundance analysis, and 200 μg was used for D isotope abundance detection. The δ^13^C and δD values in the samples were determined using an elemental analysis-stable isotope ratio mass spectrometer in combination with a Vario PYRO Cube high-temperature pyrolysis organic element analyzer (Elementar, Langenselbold, Hesse, Germany) and an Isoprime 100 isotope mass spectrometer (Isoprime, Cheadle Hulme, Greater Manchester, UK). Then, the values of ^13^Cα/^12^Cα and D_6_/H_6_ were calculated using the following formula.

^13^C/^12^C (%) = 1.089678181% × (1 + δ^13^C/1000) (1)

^13^Cα/^12^Cα (%) = 1.07252% + (^13^C/^12^C − 1.07252%)/0.2073 × 10 (2)

^13^Cγ/^12^Cγ (%) = 1.07252% + (^13^C/^12^C − 1.07252%)/0.2073 × 10 (3)

In Equations (1)–(3), ^13^C/^12^C is the ratio of ^13^C to ^12^C abundance in the sample (%). The δ^13^C is the ^13^C isotope deviation of the standard sample (Pee Dee Formation, Cretaceous, SC, USA, PDB, ‰). ^13^Cα/^12^Cα and ^13^Cγ/^12^Cγ are the isotope abundance ratios ^13^C and ^12^C of Cα and Cγ in the lignin side chain of the sample. Approximately 1.07252% is the natural abundance of ^13^C isotope in wheat straw, 0.2073 is the lignin content in wheat straw, and 10 is the ratio of the total content of ^12^C in the guaiacyl propane-type structural units to the content of ^13^Cα and ^13^Cγ.

D/H (%) = 0.015% × (1 + δD/1000)(4)

D_6_/H_6_ = 0.01317% + (D/H − 0.01317%)/0.445 × 5 (5)

In Equations (4) and (5), D/H is the isotope ratio. δD is the deviation between the D isotope abundance value of the labeled sample and the standard sample (Vienna Standard Mean Seawater, VSMOW, ‰). D_6_/H_6_ is the abundance ratio of D to H in glucose 6-C during sample preparation. Approximately 0.01317% is the natural abundance of the D isotope in wheat straw, 0.445 is the cellulose content in wheat straw, and 5 is the ratio of the total content of H in the glucose unit to the D content in glucose 6-C.

#### 3.2.5. Determination of Cross-Polarization/Magic Angle Spinning (CP/MAS) ^13^C-NMR Spectrum of Wheat Internode Tissue Powder

A Bruker AVANCE III HD 400 MHz wide-cavity solid-state NMR spectrometer (Bruker BioSpin GmbH, Ettlingen, Germany) was used, and the samples were scanned continuously 4800 times at 100 MHz using conventional CP and MAS methods. The experimental conditions were as follows: temperature: 303 K, pulse delay: 3 s, acquisition time: 0.0127 s, and pulse width: 40 kHz.

#### 3.2.6. Preparation of Kraft Pulp (KP) Cellulose–Lignin Complex (KP-CLC) and KP Xylan–Lignin Complex (KP-XLC) from KP

##### Kraft Cooking Process

**Figure 12:**
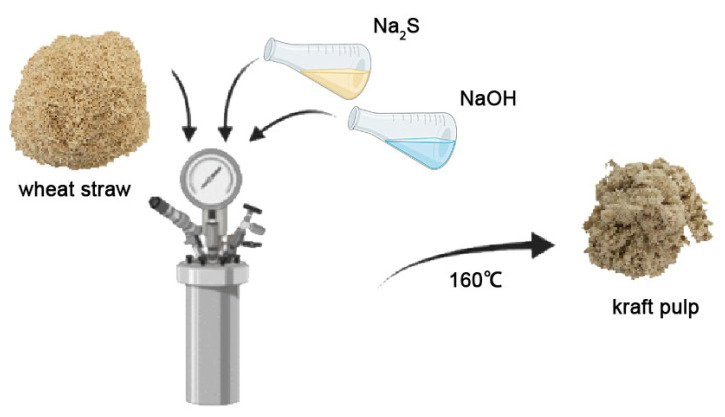
Digestion process of wheat straw kraft pulp.

The cooking process is illustrated in Figure 12. Air-dried wheat straw was milled to a 60–80 mesh size using a Weily mill. Wheat stalk powder (15 g) was cooked using the kraft method. The cooking conditions were as follows: straw-to-liquid ratio: 1:7, active alkali charge: 17% (Na_2_S), sulfidity: 25%, maximum temperature: 160 °C, cooking time: 1 h. After cooking, the pulp rich in CLC and LHC was obtained through washing.

##### Determination of Klason Lignin Content of KP

Next, 2 g KP (O.D.) was placed in a 250 mL Erlenmeyer flask. Then, 40 mL 72% sulfuric acid was added, and we kept it at 20 °C for 2 h with shaking. Then, it was washed in a 2 L Erlenmeyer flask, distilled water was added to a total volume of 1540 mL, and the mixture was boiled in an electric furnace for 4 h. During this period, water was added continuously to maintain a constant total volume. Crude Klason lignin was obtained via filtration through a G4 glass filter, and the difference in weight before and after filtration was the mass of the crude Klason lignin, *m*_1_. The residue was first carbonized in an electric furnace and then moved into a muffle furnace at 575 °C for 4 h. The difference before and after burning was measured as the mass *m*_2_ of ash in the Klason lignin. The Klason lignin content *X* (%) in the wheat straw KP was calculated using Equation (6):

(6) $$X=\frac{m_{1}-m_{2}}{m_{0}}\times 100\%$$

In the equations, *m*_0_ is the mass of wheat straw kraft pulp (O.D.) (g), *m*_1_ is the mass of acid-insoluble lignin residue (g), and *m*_2_ is the mass of ash in acid-insoluble lignin (g). The error between the two values did not exceed 0.20%. After kraft pulping, lignin and carbohydrates were largely removed, while the LCC-rich component was retained, which had practical importance for our subsequent study of the structure of LCC.

##### Classification of KP Using Ionic Liquid

Next, 2 g of dry KP (O.D.) was completely dissolved in a 20 mL (1/1, *v*/*v*) solution of DMSO/TBAH under magnetic stirring for 24 h. The solution was slowly added dropwise to 200 mL of deionized water with stirring, and then centrifuged. The resulting precipitate was washed with deionized water to neutralize, and freeze-dried at −80 °C to obtain the KP-CLC. The supernatant was magnetically stirred with dilute hydrochloric acid to adjust the pH to neutral. Then, it was dialyzed using a membrane with 1 kilo dolton (KDa) cut molecular weight and freeze-dried at −80 °C to obtain the KP-XLC.

#### 3.2.7. Determination of KP and KP-CLC Using an X-ray Diffractometer (XRD)

Then, 0.5 g of wheat KP and KP-CLC powder (O.D.) was scanned with a German Bruker D8 XRD (Bruker BioSpin GmbH, Ettlingen, Germany). The scanning range was 5–40° and the scanning speed was 5°/min.

#### 3.2.8. Enzymatic Treatment of KP-CLC and KP-XLC

**Figure 13:**
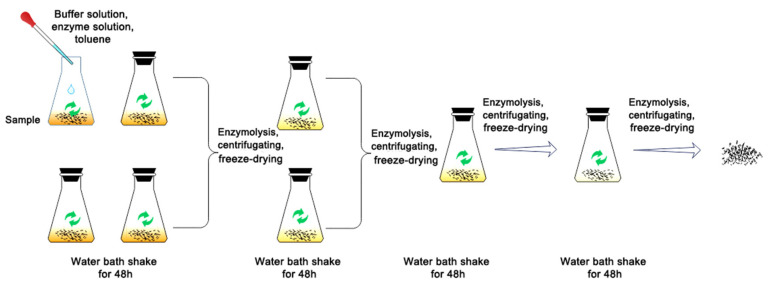
Enzymatic hydrolysis process of lignin–carbohydrate complexes.

The enzyme solution was prepared according to the following procedure: 6.56 g sodium acetate and 4.64 mL glacial acetic acid were dissolved in 4 L of deionized water to prepare a buffer solution of pH 4.5. Then, 10 g cellulase (Onozuka RS, Yakult Pharmaceutical Industry Co., Ltd. Tokyo, Japan), which mainly contains endo-β-glucanase and β-glucosidase, with a total activity ≥ 16,000 U/g) [37] was dissolved in 100 mL sodium acetate/acetic acid buffer solution, and a G2 glass filter was used to remove insoluble impurities. As shown in Figure 13, 2 g of KP-CLC samples was added to 4 (100 mL each) Erlenmeyer flasks, each containing 0.5 g samples, 1.2 mL enzyme solution, 40 mL buffer solution, and 2 drops of toluene as protective agent [38,39], and those were treated in a 45 °C water bath shaker for 48 h. After centrifugation, the residues in the Erlenmeyer flasks were collected, the four bottles were combined into two bottles, and the same enzymatic hydrolysis conditions were used for re-enzymatic hydrolysis. The residue in the bottle was collected via centrifugation and the two bottles were combined into one bottle. The same enzymatic hydrolysis conditions were used. The fourth enzymatic hydrolysis step was performed with a 0.6 mL enzyme solution under the same conditions. After each enzymatic hydrolysis, the residues were washed with diluted HCl of pH 3 three times and centrifuged and freeze-dried to obtain an enzymatically degraded cellulose–lignin complex (En-KP-CLC).

The procedure for hydrolysis of the KP-XLC fraction was as follows: 1 g of xylanase mainly containing β-1,4-xylanase and β-xylosidase, with total activity ≥ 6000 U/mg and 1 g of hemicellulose (from *Aspergillus niger*, Sigma-Aldrich, which mainly contains β-glucanase, galactosidase, and xylanase, with total activity ≥ 150 KU/g) were dissolved in 10 mL cellulase solution and filtered through a G2 glass filter to remove insoluble impurities. The enzymatic hydrolysis process was the same as that for the CLC, and enzymatic degradation of the xylan–lignin complex (En-KP-XLC) was achieved after four enzymatic hydrolysis cycles. The treatment process is illustrated in Figure 13.

#### 3.2.9. Acetylation of the En-KP-CLC Sample

En-KP-CLC (200 mg) was added to DMSO/N-methylimidazole (4 mL/2 mL) and the mixture was stirred for 12 h. Acetic anhydride (1.5 mL) was then added and the reaction was allowed to proceed for 2 h. After the completion of the reaction, the mixture was slowly added to 80 mL of distilled water and allowed to stand for 6 h. The mixture was then centrifuged to obtain a solid residue. The product was washed three times with deionized water and freeze-dried to obtain the acetylated product Ac-En-KP-CLC.

#### 3.2.10. Determination of NMR Spectra of Ac-En-KP-CLC and En-KP-XLC Samples

The sample (85 mg) was completely dissolved in 0.6 mL of DMSO-d6 and placed into a φ5 mm NMR tube. The sample was scanned using a BRUKER AVANCE III HD600 NMR spectrometer (Bruker BioSpin GmbH, Rheinstetten, Germany). The scanning parameters were as follows: scan frequency: 150.92 MHz, temperature: 298 K, pulse delay: 2 s, acquisition time: 0.8 s, and cumulative scan: 10,000 times.

^1^H-NMR spectrum recording parameters were as follows: scan frequency: 600 MHz, temperature: 298 K, pulse delay: 1.0 s, acquisition time: 3.14 s, and 512 scans with 12 k acquisition data points.

## 4. Conclusions

(1) The abundances of ^13^C and D in the experimental groups were substantially higher than those in the control group. These results indicate that the injected exogenous coniferin-[α-^13^C], coniferin-[γ-^13^C], and d-glucose-[6-D_2_] were effectively absorbed and metabolized during the growth of wheat internode tissues. Therefore, the lignin in the cell wall of wheat straw was labeled with ^13^C and the polysaccharides were labeled with D.
(2) CP/MAS ^13^C-NMR determination and analysis of the differential spectroscopy of wheat straw powder in the ^13^Cα-labeled experimental group, ^13^Cγ-labeled experimental group, and unlabeled control group showed that after the labeling of lignin side chain ^13^Cα and ^13^Cγ, the signals in the CP/MAS ^13^C-NMR spectra were substantially enhanced compared with that of the control group. The chemical linkages between lignin and lignin were mainly β-aryl ether, β-5, β-1, and β-β. Lignin was primarily linked to polysaccharides via acetal, benzyl ether, and benzyl ester bonds.
(3) After kraft cooking of the wheat straw, a large amount of lignin and xylan was dissolved, but most of the CLC and XLC was retained. The obtained KP was fractionated with the ionic liquid DMSO/TBAH to obtain cellulose–lignin and xylan–lignin complexes. The XRD results of the KP and KP-CLC showed that the KP-CLC was mainly converted from cellulose type I to cellulose type II in the crystalline region after ionic liquid classification.
(4) The ^13^C-NMR and ^1^H-NMR spectra of Ac-En-KP-CLC showed that the signal intensity between lignin–lignin and lignin–cellulose was substantially enhanced after the lignin side chain was labeled with ^13^Cα and ^13^Cγ. The ^13^C-NMR and ^1^H-NMR spectra of AC-En-KP-CLC showed that the CLC structure obtained after enzymatic hydrolysis by cellulase was mainly chemically bonded by acetal and benzyl ether bonds. The ^13^C-NMR spectrum of En-KP-XLC showed that the XLC structure obtained after enzymatic hydrolysis of xylanase contained some lignin side chain xylan linked to Cα by acetal and ether bonds.

## Figures and Tables

**Table 1:** ^13^C abundance of wheat straw.

	δ^13^C (‰)	^13^C/^12^C (%)	^13^Cα/^12^Cα (%)	^13^Cγ/^12^Cγ (%)
Control	−27.56	1.076	1.076	1.076
A	−1.28	1.104	2.604	-
B	7.92	1.114	-	3.089

Notes: Control: control group; Group A: injected with coniferin-[α-^13^C], d-glucose-[6-D_2_], and 4CL inhibitors; Group B: injected with coniferin-[γ-^13^C], d-glucose-[6-D_2_], and 4CL inhibitors.

**Table 2:** D abundance of wheat straw.

	δD (‰)	D/H (%)	D6/H6 (%)
Control	58.692	0.016	0.016
A	1356.568	0.037	0.280
B	1911.743	0.045	0.370

Notes: Control: control group; Group A: injected with coniferin-[α-^13^C], d-glucose-[6-D_2_], and 4CL inhibitors; Group B: injected with coniferin-[γ-^13^C], d-glucose-[6-D_2_], and 4CL inhibitors.

**Table 3:** Assignments of signal from CP/MAS ^13^C-NMR difference spectrum of wheat straw.

Signal	^13^C (ppm)	Assignments
1	109.0	Cα in lignin linked to carbohydrate by acetal bond
2	82–88	Cα in lignin linked to carbohydrate by benzyl ether bond, Cα in lignin β-5, β-β structure
3	76.4	Cα in lignin linked to carbohydrates by benzyl ester bond
4	74.6	Cα in β-O-4 structure
1′	167.3	Cγ of ferulic acid derivatives
2′	72.6	Cγ in β-β structure of lignin
3′	64.4	Cγ in lignin β-5
4′	62.5	Cγ in β-O-4 and β-1 structure of lignin

**Table 4:** Concentrations and compositions of the solutions injected into wheat stalks.

	Coniferin-[α-^13^C]	Coniferin-[γ-^13^C]	d-Glucose-[6-D_2_]	4CL Inhibitor
Control	-	-	-	-
Group A	5 mg/mL	-	3.33 mg/mL	4 mg/mL
Group B	-	5 mg/mL	3.33 mg/mL	4 mg/mL

Notes: Group A: injected coniferin-[α-^13^C], d-glucose-[6-D_2_], and 4CL inhibitors; Group B: injected coniferin-[γ-^13^C], d-glucose-[6-D_2_], and 4CL inhibitors; control: intact wheat.

## Data Availability

The data presented in this study are available in the manuscript.
